# A Bayesian Small Area Estimation Approach for District‐Level Fertility and Mortality Estimates in India, 2015–16 to 2019–21

**DOI:** 10.1002/hsr2.71789

**Published:** 2026-02-08

**Authors:** Laxmi Kant Dwivedi, Somnath Jana, Shekhar Chauhan, Pooja Arora, Priyanka Dixit, Mrigesh Bhatia

**Affiliations:** ^1^ Department of Survey Research & Data Analytics International Institute for Population Sciences Mumbai India; ^2^ International Institute for Population Sciences Mumbai India; ^3^ Claude Pepper Center, Florida State University; ^4^ School of Health Systems Studies Tata Institute for Social Sciences (TISS) Mumbai India; ^5^ Department of Health Policy London School of Economics London UK

**Keywords:** bayesian, district‐level, India, infant mortality rate, NFHS‐4 & NFHS‐5, neonatal mortality rate, small area estimation, total fertility rate, under‐five mortality rate

## Abstract

**Background and Aims:**

Fertility and child mortality are critical public health indicators in India, directly influencing health policy, planning, and intervention effectiveness. The relationship between declining fertility and decreasing child mortality has been widely debated. This study aims to estimate and compare fertility and child mortality rates at the district level using data from the National Family Health Survey (NFHS) rounds 4 and 5, with a focus on understanding regional trends and their implications for health interventions.

**Methods:**

The study investigates four key demographic indicators: Total Fertility Rate (TFR), Neonatal Mortality Rate (NMR), Infant Mortality Rate (IMR), and Under‐Five Mortality Rate (U5MR). Fertility and child mortality rates were estimated using Bayesian methods, aligned with Demographic and Health Survey (DHS) standards. Fertility rates were computed in two stages: first, birth history data were transformed into table of birth, followed by the calculation of fertility rates through Poisson regression models at the district level.

**Results:**

Between 2015–16 and 2019–21, the number of districts with a TFR below 1.6 increased from 21 to 166, while number of districts with a TFR between 1.6 and 2.1 remained stable at 328. Additionally, the number of districts with an NMR under 10 per 1,000 live births grew from 79 to 140. The study found a strong association between the reduction in child mortality and the decline in fertility rates.

**Conclusion:**

This study suggests that addressing regional variations in fertility and child mortality rates could enhance the effectiveness of health interventions in India. Policymakers should prioritize expanding access to family planning and maternal‐child health services. The availability of district‐level data will support more targeted and effective health policies tailored to local needs.

## 1 Introduction

Family planning policies in India have been in discussion for a long. Despite having the vision to limit fertility rates by adopting family planning methods since the beginning, India failed to achieve the required targets. During the infamous emergency period of 1975‐77, an increase in sterilization was observed in India [1]. Relying on the ideology of ‘development is the best contraceptive,’ many scholars have associated improvements in maternal and child health services, specifically reduction in infant and child mortality, to reduced fertility rates across regions. Conversely, higher fertility rates have also been linked to higher infant and child mortality [2]. Demographic transition theory suggested that only as couples became richer and better educated would they choose to have fewer children. Various plausible mechanisms are underlying the relationship between fertility and mortality rates. However, the most renounced of them all is that declining child mortality would reduce fertility levels. Recent debates on population control have renewed calls for a comprehensive population policy to reduce fertility levels in India, while also questioning the need for a two‐child policy and arguing that such a norm should be enforced only after all other population control measures have been exhausted. Since the total fertility rate (TFR) is nearing replacement level in almost majority of the states in India, is it worth promoting a two‐child policy in India, or further development in child mortality indicators would automatically harness the desired results of controlling fertility levels is a question that needs an answer. In this study, we seek to examine the concordance of child mortality indicators with fertility indicators to confirm whether or not the decline in total fertility rate (TFR) is parallel to the decline in various mortality indicators across districts in India from 2015–16 to 2019–21.

Infant and child mortality rates are widely used indicators to envisage the development achieved by a nation. The sensitivity of those mortality indicators can be experienced through an extensive range of demographic, socio‐economic, and environmental factors, which are deeply driven in society and require massive effort to yield a population change. India is known to contribute significantly to the global burden of child death, with a staggering size of 1.2 million death occurring among under‐five children in 2015 [3]. Estimates suggest that the districts lagging in achieving the targets for development indicators are mainly lagging by neo‐natal mortality rates (NMR) [4]. In fact, there is an increase in the NMR during 1992–2016 in India [5]. Changes in child mortality are directly influenced by improvements in maternal and child health and poverty status in India, which directs the fertility change in the population. Improvement in child survival increases the likelihood for fertility choice towards a smaller number of children. Improvement in Total Fertility Rate (TFR) over the years has fundamentally occurred due to structural changes such as demographic characteristics and behavioural change in a population [6]. To delve deeper into the individual factors, one has to highlight the role played by the factors like education, work participation, usage of contraceptives, access to basic healthcare services, etc., which supposedly impacted the population through multiple, interlinked and coherent programmes and policies implemented by the government and relevant stakeholders. The outcome of these development processes could not be placed in a single platform since regional heterogeneity and spatial dispersion are pretty visible [7]. Variations in child mortality exist at different level of fertility; the demographic segregation of the level of fertility is defined through different schools of thought. However, the convergence theory for fertility would ultimately evoke the decline in fertility of the states with a different pace to be specific [8]. The divergence in fertility outcome across the major states during 1981‐91 marks that southern states have realized a higher decline in fertility than northern states of India.

The decline in spatial heterogeneity in fertility is a matter of temporal changes that occurred and the speed of demographic transitions realized by the region [7]. The endowment of the inherent socio‐cultural factors related to reproductive and child health is not deniable; however, the impetus realized from the programmatic approach to ameliorate those sociocultural factors needed to be scrutinized in this context. The socio‐cultural and economic evaluation of the unwanted child is vital to determine the replacement effect of children in terms of relative fertility. If the states experience high child mortality, the perception towards mortality decline in the community would hardly affect the fertility choice of the population. The response to hoarding of children in high mortality states like Uttar Pradesh affects child birth minimally compared to Karnataka. Therefore, a decline in fertility would be higher in Karnataka, where child mortality shows a significant decline and economic values attached to the children is effectively greater [9]. The lower sex displacement also determines the replacement effect of fertility. A state like Uttar Pradesh would not prefer to replace a male child with a female if the previous child did not survive of a similar sex.

Linking the community‐level perception can be heavily reflected by the programmes and policies applied in that particular context. Engaging the community through the Panchayati Raj system and other stakeholders, including concerned ministries, has been evoked in the last few decades to adequately address community‐specific demands in the development policies. As oldest programmes like Integrated Child Development Schemes (ICDS), in conjunction with other nutrition, health, and women‐related programmes and policies, are relentlessly trying to improve health and fertility outcomes, especially in the backward districts of India [10]. To understand the dynamics of fertility decline, it is essential to understand the trickling‐down effect of the developmental processes, which also differ across spaces. A persistent differential in sex, rural‐urban, caste, religion, educational and economic groups already exist [11, 12, 13, 14]. Even though the vertical diffusion of the behaviour for fertility decline is more pronounced in the advanced groups of states, the differential effects among socio‐economic subgroups are still found within those states. Additionally, a good population and health policy demands technical support that can be easily measured and might avoid indirect estimation for child survival, per se. This study aims to provide the estimates and changes in key child mortality indicators namely, Neonatal Mortality Rate (NMR), Infant Mortality Rate (IMR), and Under‐Five Mortality Rate (U5MR) in relation to the levels of fertility, measured by the Total Fertility Rate (TFR), across Indian districts between 2015–16 and 2019–21. The resulting estimates will provide insights into the spatial patterns of fertility and child mortality, thereby contributing to the assessment of the effectiveness of existing health programmes and policies in India.

## 2 Data and Methods

A population‐based cross‐sectional dataset has been used in the study from the Indian version of Demographic and Health Survey (DHS), the National Family Health Survey (NFHS‐4), 2015–16 and NFHS‐5, 2019–21, which are publicly available. NFHS‐4 focuses on the 640 districts in all 29 states and seven union territories and NFHS‐5 studies 707 districts of India in all 28 states and eight union territories. The NFHS‐4 survey has a sample of 6,01,509 households and 6,99,686 women aged 15–49 years, whereas, NFHS‐5 has 6,36,699 households and 7,24,115 women aged 15–49 years. The NFHS survey sample was designed to produce health and demographic indicators for all the selected districts of India. Information about the birth history and child mortality was collected from each interviewed ever married woman. This allows for fertility and child mortality indicators to be calculated from the survey data.

The analysis used data from the National Family Health Survey (NFHS‐5), a nationally representative, two‐stage stratified sample covering all districts of India. No districts or regions were specifically targeted or excluded. The NFHS employs a uniform sampling design to ensure representativeness at the national, state, and district levels. Regarding missing data, cases with incomplete birth history information relevant to fertility estimation were excluded from the analysis following DHS guidelines. No imputation was performed, as the proportion of missing data was minimal and unlikely to bias the results.

### 2.1 Outcomes

The study has focused on major indicators of fertility and mortality; those are Total Fertility Rate (TFR), the Neonatal Mortality Rate (NMR), the Infant Mortality Rate (IMR), and the Under‐five Mortality Rate (U5MR). The four indicators are defined and calculated according to the DHS approach [15]. TFR is the average number of births a woman would have at the end of her childbearing period if she passed through this period bearing children at observed age‐specific fertility rates. Similar to other Demographic Health Survey (DHS) surveys, in the NFHS‐4, TFR is calculated for the 3 years before the survey based on detailed birth histories provided by women. All the child mortality rates are calculated for the 5 years prior to the survey, based on women's detailed birth and death histories as per the DHS statistical guidelines [16].

## 3 Methodology

### 3.1 Methodology to Compute Child Mortality Rates

We have used Bayesian approaches to determine the child mortality rate using DHS data. The numerator is defined as the number of deaths of live‐born children within a specific age range and specified period. The child mortality rate is determined as the quotient of the numerator divided by the denominator for each kind. Fatalities first 28 days old are considered when calculating NMR. Similarly, the U5MR is assessed at ages 0–5 years, including fatalities recorded at ages 0–59 months, while the IMR is measured at ages 0–11 months. The denominator is the number of children living at the start of the selected age range throughout the defined period [17]. We use Monte Carlo Markov chain (MCMC) techniques to get Bayesian estimates.

### 3.2 Methodology of Estimating Fertility Rates

Here we use two steps to calculate the fertility rates. Firstly, a Stata code to transform the birth history data in a table of birth. Secondly, we use Poisson regression to compute the fertility rates from birth history data [18]. More Specifically,

Let Xi be the random variable denotes the number of birth (xi be the realization of Xi) is assumes to follow a Poisson distribution with mean *μi*

(1) $$P(\text{xi}=\text{xi|}\mu i)=\frac{\text{exp}\left(\mu i\right)*\mu \text{i\textasciicircum{}xi}}{\mathrm{xi}!}$$

the mean *μi*=(fertility rate (*λ*i)*exposure (ti), now taking log over it, we have

(2) log(μi)=log(λi)+log(ti)

log(*λ*i) is a linear combination of independent variables; thus

(3) $$\log(\lambda i)=\alpha +f_{1}(\text{age})+f_{2}(\text{covariates})$$

now for 5 years age group, we make dummy variables in the form.

$$\log\left(\mu_{i}\right)=\log\left(t_{i}\right)+\left(\alpha +\sum_{w=20-24}^{45-49}\beta_{w}A_{\mathrm{wi}}\right)$$

α is the constant term; *A*w*i* are dummy variables for the six age groups from 20 to 24 to 45–49, and we have the first age group (15–19) as the reference category.

So, the fertility rate $(\lambda i)=\text{exp}\;(\alpha +\sum_{w=20-24}^{45-49}\beta \text{wAwi}$)

predicting fertility rates for a specific age group (e.g., 20–24 years) is straightforward. The dummy variable A equals 1 in the particular age group and 0 for the other age groups; the rate is similar to the exponential of the constant and corresponding age groups’ coefficient (20–24).

$$\lambda_{20-24}=[\alpha +\beta_{20-24}]$$

$$\mathrm{TFR}=5*[\text{exp}\left(\alpha \right)+(\text{exp}\left(\alpha +\sum_{\omega =20-24}^{45-49}\beta_{\omega }\right)]$$

### 3.3 Bayesian Approach and Simulation

Various models for estimation are available based on our requirements and the availability of auxiliary information. The hierarchical Bayesian (HB) method was implemented using the Gibbs sampling approach. In the HB model, the priors for hyperparameters (model parameters) are also defined in addition to the prior parameters, and then the inference is made based on the posterior distributions. In the logistic‐normal mixed model, the sampling variances are treated as unknown parameters and are estimated from the posterior distribution during model fitting.

We generated a Markov chain of random states and retained every k‐th draw after the burn‐in period to reduce autocorrelation and obtain approximately independent samples. Since the initial simulated states cannot be used as samples, we refer to the time it takes to attain stationarity as the “burn‐in time”. In reality, the examination of the autocorrelation function may be used to determine the latency necessary for two states to be regarded as nearly independent.

Additionally, to enhance the precision of district‐level estimates and capture the true local demographic patterns, we applied a Small Area Estimation (SAE) technique. This approach uses a link function to statistically relate survey‐based estimates with auxiliary information from the Census‐2011 (Primary Census Abstract District level file), allowing for more reliable predictions in areas with limited sample sizes in survey data [19]. Key auxiliary variables incorporated into the model included average household size, percentages of Scheduled Caste and Scheduled Tribe population, female literacy rates indicators known to influence fertility and child mortality patterns. By borrowing strength from these contextual variables, the SAE approach helps stabilize estimates at the district levels [20]. This method ensures that the estimates not only reflect sample observations but also the broader socio‐economic environment of each district.

## 4 Results

### 4.1 Prevalence Maps and Tables

**Figure 1:**
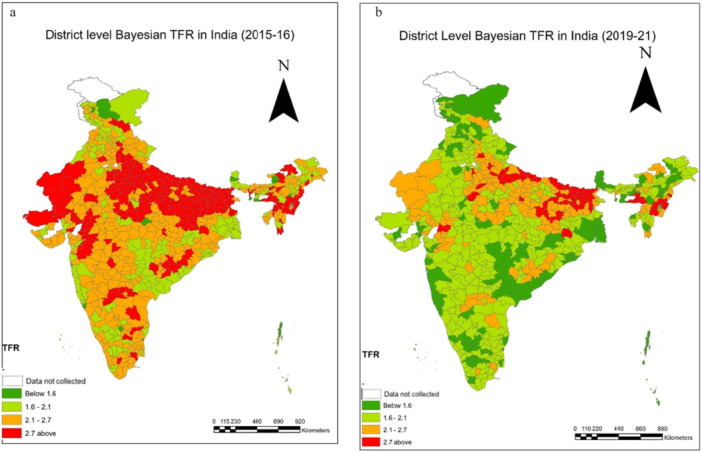
a, b: District level estimates of Total Fertility Rate (TFR) using Bayesian Method during 2015‐16 to 2019‐21.

The results illustrate the shifts in both child mortality and fertility indicators over time (Tables 1, 2, 3, 4). In the case of the total fertility rate (TFR), it is observed that in 2015–16, there were only 21 districts with a TFR < 1·6 (Table 1), but in 2019–21, the number of these districts climbed to 166 (Table 1). There is not much variation between the two‐time points in the fertility rate range of 1.6–2.1 (Figure 1a,b); in 2015–16, there were 322 districts, and in 2019–21, there were 328 districts (Table 1). Interestingly, there has been a decline in the number of districts for TFR over 2.7; the statistics reveal that in 2015–16, 136 districts had a TFR above 2.7, but in 2019–21, that number dropped to just 66. A decrease in TFR may be seen in the prevalence map also which reveals a concentration of high TFR in northwestern and eastern areas (Figure 1a,b). In 2015–16, districts in Rajasthan, Bihar, and Uttar Pradesh had higher TFR (Supporting Information: Tables 5 and 6). In the transition to earlier findings, we also observed clustering of TFR between 1.6 and 2.1 in the period 2019–21 (Figure 1a,b).

**Table 1:** District‐level transition of Total Fertility Rate during 2015–‐16 to 2019–21.

TFR	NFHS 4		NFHS 5	
	*n*	%	*n*	%
< 1.6	21	3.28	166	23.48
1.6–2.1	322	50.31	328	46.39
2.1–2.7	161	25.16	146	20.65
Above 2.7	136	21.25	67	9.34
Total	640		707	

**Table 2:** District‐level transition of Neonatal Mortality Rate during 2015–16 to 2019–21.

NMR	NFHS 4		NFHS 5	
	*n*	%	*n*	%
< 10	79	12.34	140	19.8
10.01–20.00	138	21.56	212	29.99
20.01–30.00	159	24.84	169	23.9
30.01–40.00	146	22.81	111	15.7
40.01 and above	118	18.44	75	10.61
Total	640		707	

**Table 3:** District‐level transition of Infant Mortality Rate during 2015–16 to 2019–21.

IMR	NFHS 4		NFHS 5	
	*n*	%	*n*	%
< 15	66	10.31	115	16.27
15.01–25.00	101	15.78	161	22.77
25.01–35.00	115	17.97	162	22.91
35.01–45.00	128	20	126	17.82
45.01 and above	230	35.94	143	20.23
Total	640		707	

**Table 4:** District‐level transition of Under 5 Mortality Rate during 2015–16 to 2019–21.

U5MR	NFHS 4		NFHS 5	
	*n*	%	*n*	%
< 20	76	11.88	134	18.95
20.01–35.00	143	22.34	218	30.83
35.01–45.00	103	16.09	132	18.67
45.01–55.00	97	15.16	89	12.59
55.01 and above	221	34.53	134	18.95
Total	640		707	

**Figure 2:**
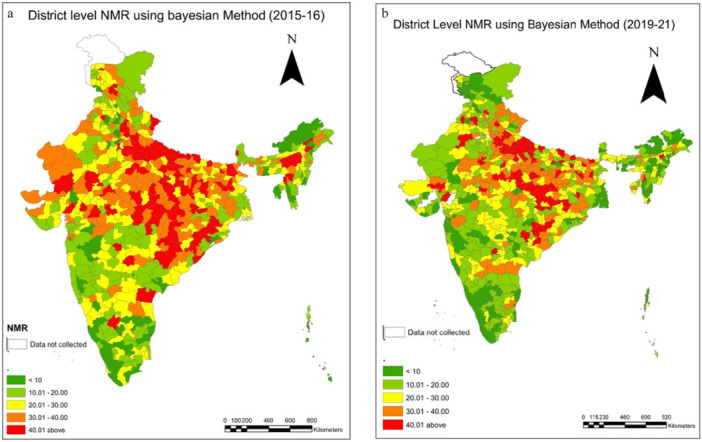
a, b: District level estimates of Neonatal Mortality Rates(NMR) using Bayesian Method during 2015–16 to 2019–21.

All mortality indicators are presented per 1000 live births. In 2015–16, there were only 79 districts with an NMR below 10, but in 2019–21, we observed 140 districts with an NMR below 10, which is a sign of favourable child health at the district level (Table 2). Moreover, the prevalence maps and corresponding results indicate a distinct clustering of NMR in the 10–20 range during 2019–21 (Figure 2a,b). Districts with an IMR exceeding 40 accounted for a substantial 18.5% (118) districts in 2015–16; however, this proportion declined markedly to 10.6% (75) districts in 2019–21 (Table 2). A pronounced concentration of high NMR was observed in the central and eastern regions of the country, particularly across Uttar Pradesh, Bihar, Madhya Pradesh, Chhattisgarh, and Odisha (Supporting Information: Tables 5 and 6). The prevalence map across two time periods also visualizes the decline in NMR (Figure 2a,b). The neonatal mortality rates have performed better in the southern and north‐eastern states, and it performed worse in India's central and northern states. Most of the districts representing a high NMR are clustered in the Upper and middle Ganga region of Uttar Pradesh. For 2015–16, the lowest IMR value is shown in the districts of Kerala (Supporting Information: Table 5,6). The highest IMR value is seen in districts of Odisha (Supporting Information: Tables 5 and 6).

Similarly, we observe changes over the two time periods in IMR and U5MR as depicted in Tables 3 and 4. It is supported by the prevalence maps (Figures 7a,b and 12a,b) of IMR and U5MR at district level over two time periods, respectively. We observed a comparable trend for Infant mortality rate (IMR) and under five mortality rates. In the case of IMR, just 10% of districts had IMRs below 15 in 2015–16; however, as health systems improved over time, it is observed that 17% of all districts had IMRs below 15 in 2019–21 (Table 3). We noticed a grouping of 23% of districts (162) with IMR for 2019–21 in the categories (25–35). The trend was also seen for the extreme ranges; in 2015–16, 36% of all districts had IMRs over 45; by 2019–21, however, that figure had dropped to 20% (Table 3). A change can also be seen in the prevalence map over a two‐time period, which shows that the IMR in the districts in southern India is lower (Figure 7a,b). As there are almost equal numbers of districts in these two categories (Table 3), we may anticipate a movement between the two groups over time. Moreover, examining the magnitude of infant mortality rates, we find evidence that the rates primarily fall between 20 and 35. Our findings reveal a shift in the U5MR over two periods (Figure 12a,b). The change is more evident in the extreme ranges, below 20 and over 55, and clustering can be seen in the prevalence range of 20 to 30 (Table 4, Figure 12B). In 2015–16, only 12% of districts had a U5MR of less than 20, but by 2019–21, the percentage had climbed to 19% (Table 4). We observed a grouping of 31% of districts (218) in 2019–21 with IMR ranges (20‐ 35) (Table 4). According to the prevalence map, the country's central, northwestern, and eastern parts are concentrated in the high‐prevalence districts (Figure 12a,b). The change in U5MR is most clearly seen in cases of high prevalence; in 2015–16, 35% of districts had U5MRs above 55; by 2019–21, that number had dropped to 19% (Table 4). For 2015–16, the distribution of U5MR suggests that around 61% (388) of districts have 55 and above under 5 mortality rates in India (Figure 12A). For both periods, the pattern of the U5MR shows no exceptions too. The lowest U5MR is again shown by the districts of Kerala and the highest by Uttar Pradesh (Supporting Information: Tables 5 and 6) (Figure 12a,b).

### 4.2 Scatter Plots

According to the rates of child mortality, scatter plots are created for various categories of TFR ranges. TFR are divided into four categories, and we compare how the TFR and child mortality rates have changed across two‐time intervals.

### 4.3 TFR Vs NMR

**Figure 3:**
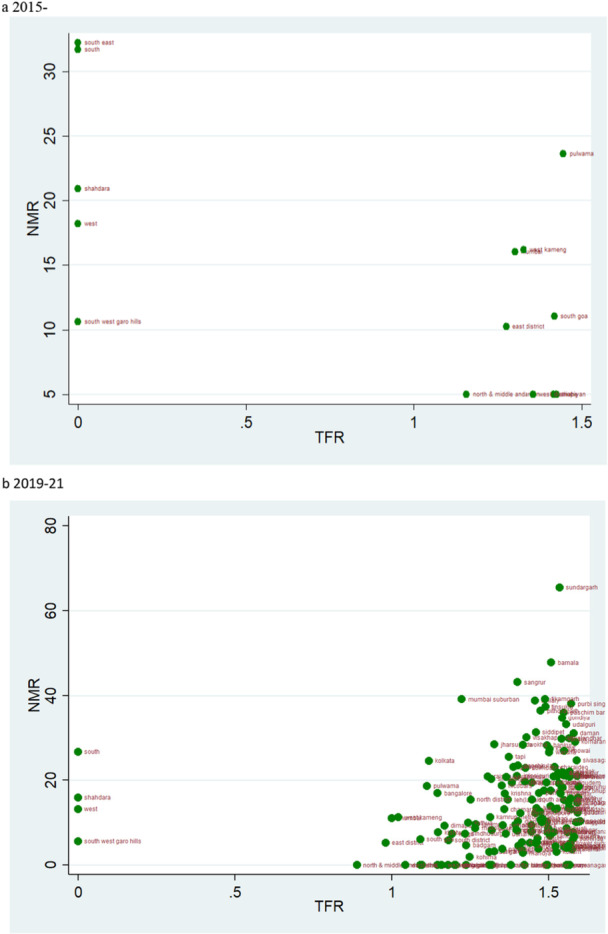
a, b: Scatter plot of district‐level Neonatal Mortality Rates (NMR) against Total Fertility Rates (TFR) for districts with TFR below 1.6 during 2015–16 to 2019–2. a 2015‐2016. b 2019–21.

The scatter plot hints at the pattern of change in NMR over the two‐time intervals in relation to different TFR levels. The data also show a clustering at the extreme two groups (Figure 3a,b and 6a,b). The scatter plot of TFR below 1.6 shows that the number of districts is higher in the years 2019–21 compared to 2015–16, which describes an improvement in NMR. Also, the ranges of NMR in these categories mostly lie below 20 (Figure 3a,b). Though the overall level of mortality estimates across the districts of these states is performing well, few outliers are there within the state behaving poorer on those indicators despite being at a similar level of fertility. In Kerala, districts like Alappuzha, Wayanad, and Kollam are showing relatively higher level of child mortality rates. The district of Western Sikkim is estimated to show relatively higher rates of child mortality (Figure 3a).

**Figure 4:**
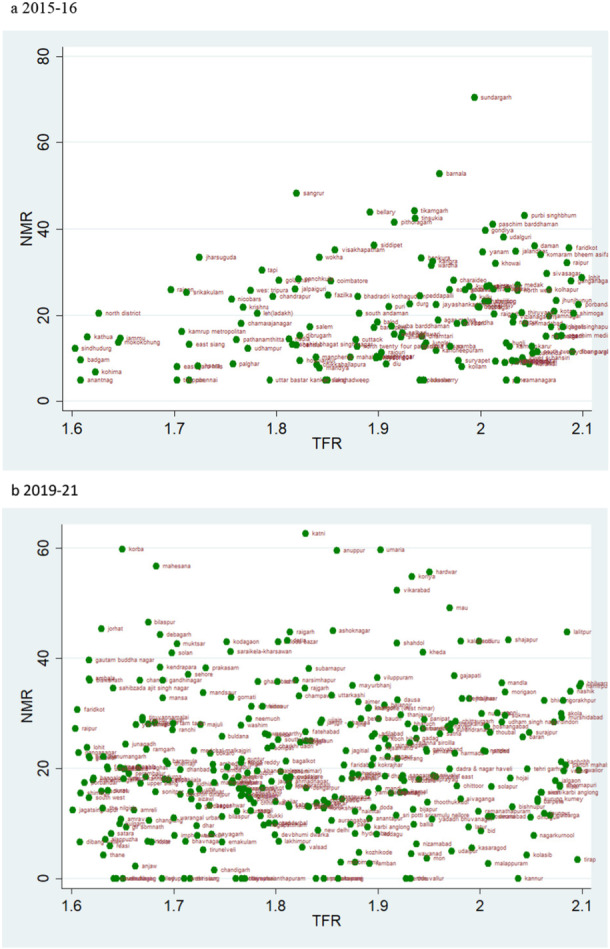
a, b: Scatter Plot of District‐Level Neonatal Mortality Rates (NMR) and Total Fertility Rates (TFR) for TFR Between 1.6 and 2.1, 2015–16 to 2019–21.

**Figure 5:**
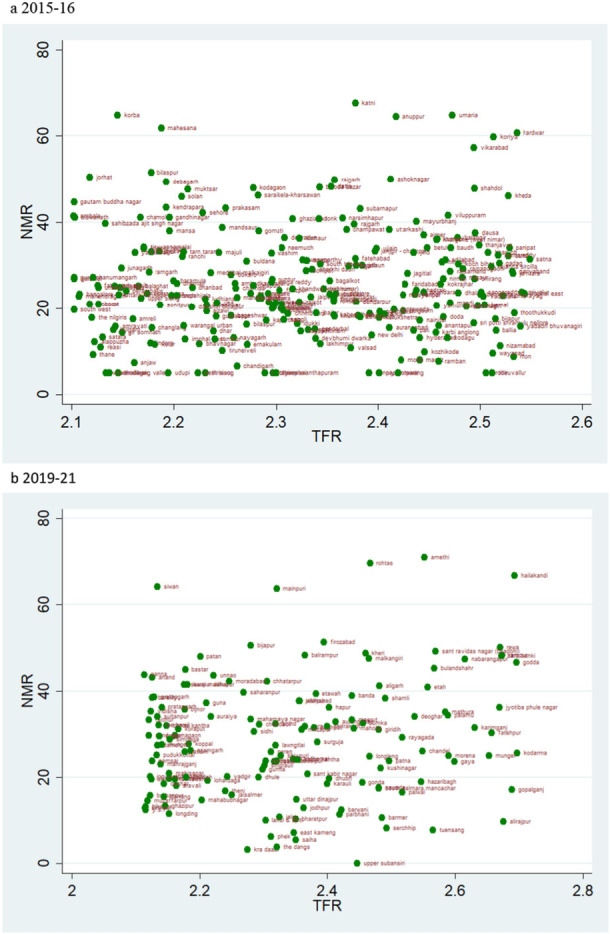
a, b: Scatter Plot of District‐Level Neonatal Mortality Rates (NMR) and Total Fertility Rates (TFR) for TFR between 2.1 and 2.7, 2015–16 to 2019–21.

**Figure 6:**
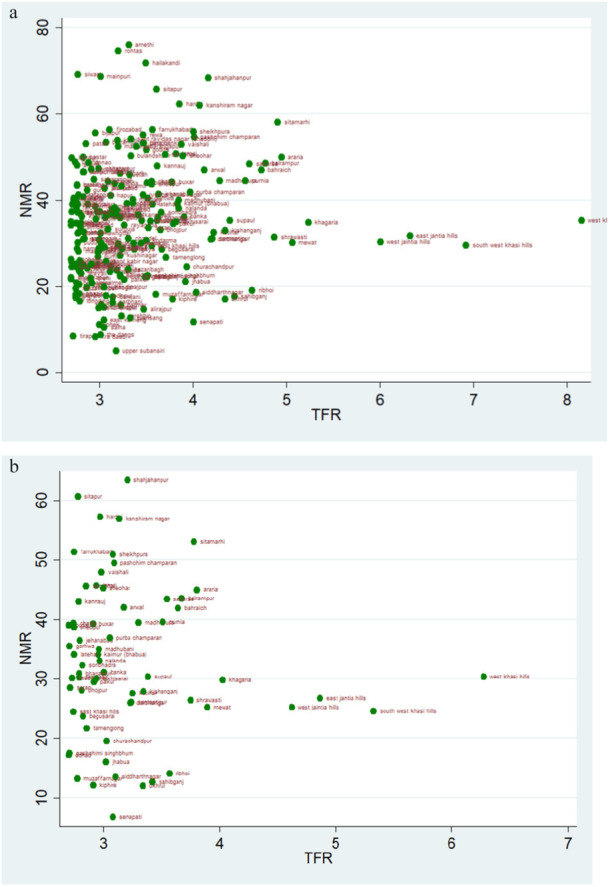
b, a: Scatter Plot of District‐Level Neonatal Mortality Rates (NMR) and Total Fertility Rates (TFR) for Districts with TFR Above 2.7, 2015–16 to 2019–21.

The magnitude of the NMR makes the sole difference in the scatterplots of TFR range 1.6–2.1 and 2.1–2.7 for the two‐time points (Figures 4a,b and 5a,b). The scatter plot of TFR over 2·7 across two‐time points shows a glaring contrast; more districts had high NMR in 2015–16 than in 2019–21 (Figure 6a,b). At the same time, Tamil Nadu is an exemplary state for its public health system and child health outcomes. For 2015–16, districts like Tiruvallur (TFR 1.5) and the Nilgiris (TFR 1.6), despite belonging to similar fertility levels, Tiruvallur shows a very high NMR, IMR, and U5MR in comparison to Nilgiris.

**Figure 7:**
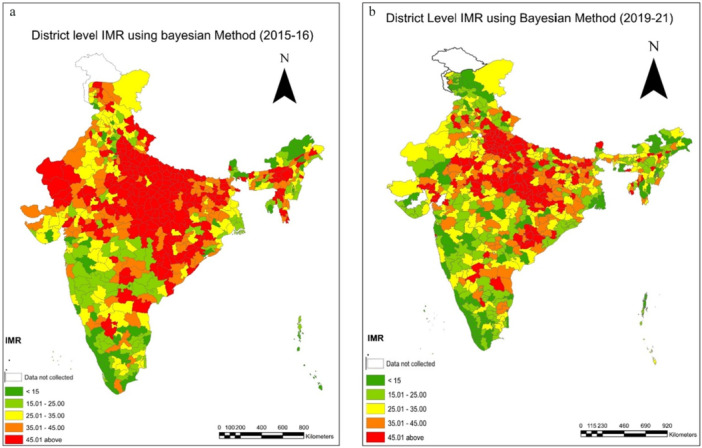
a, b: District level estimates of Infant Mortality Rates(IMR) using Bayesian Method during 2015–16 to 2019–21.

### 4.4 TFR Vs IMR

**Figure 8:**
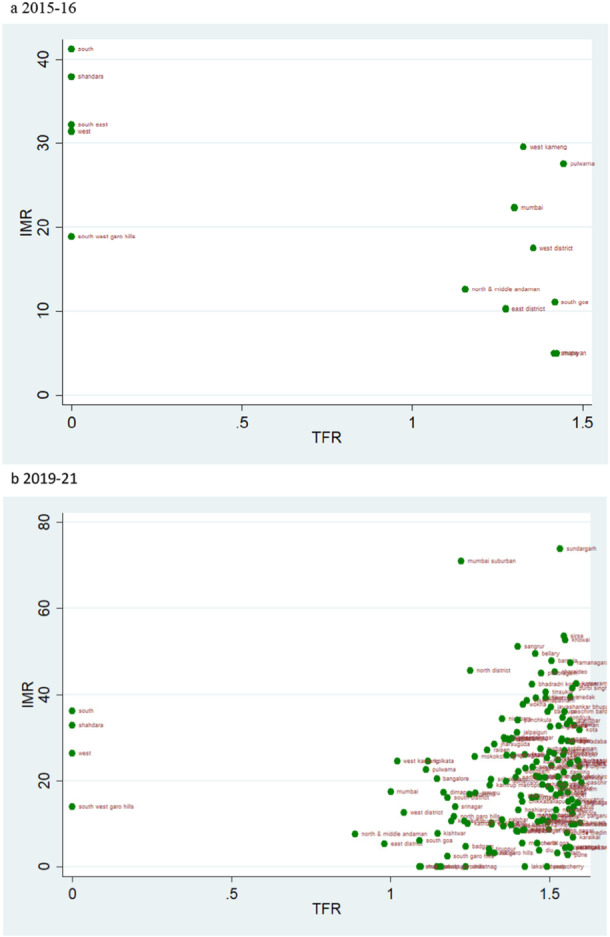
a, b: Scatter Plot of District‐Level Infant Mortality Rates (IMR) and Total Fertility Rates (TFR) for Districts with TFR Below 1.6, 2015–16 to 2019–21.

**Figure 9:**
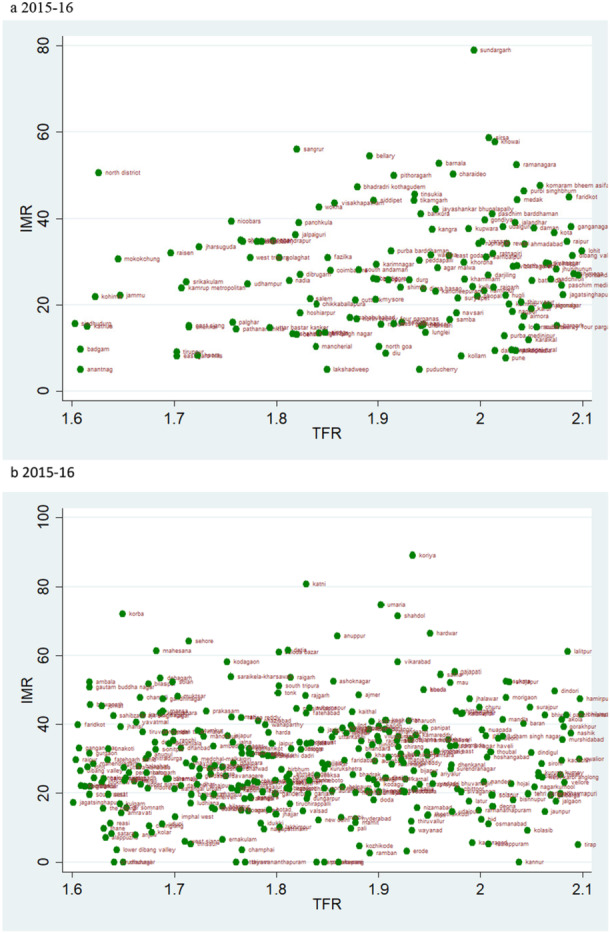
a, b: Scatter Plot of District‐Level Infant Mortality Rates (IMR) and Total Fertility Rates (TFR) for TFR Between 1.6 and 2.1, 2015–16 to 2019–21.

**Figure 10:**
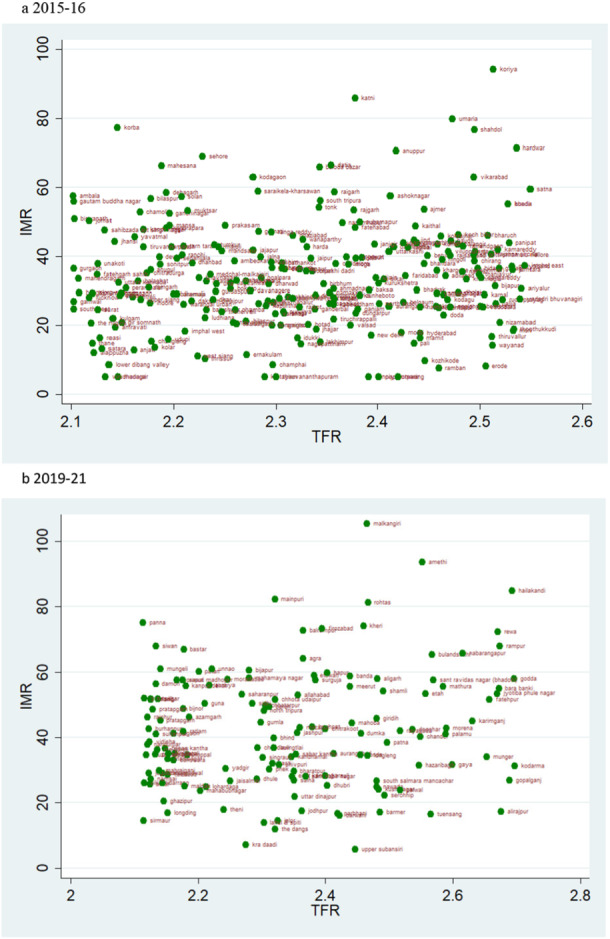
a, b: Scatter Plot of District‐Level Infant Mortality Rates (IMR) and Total Fertility Rates (TFR) for TFR Between 2.1 and 2.7, 2015–16 to 2019–21.

**Figure 11:**
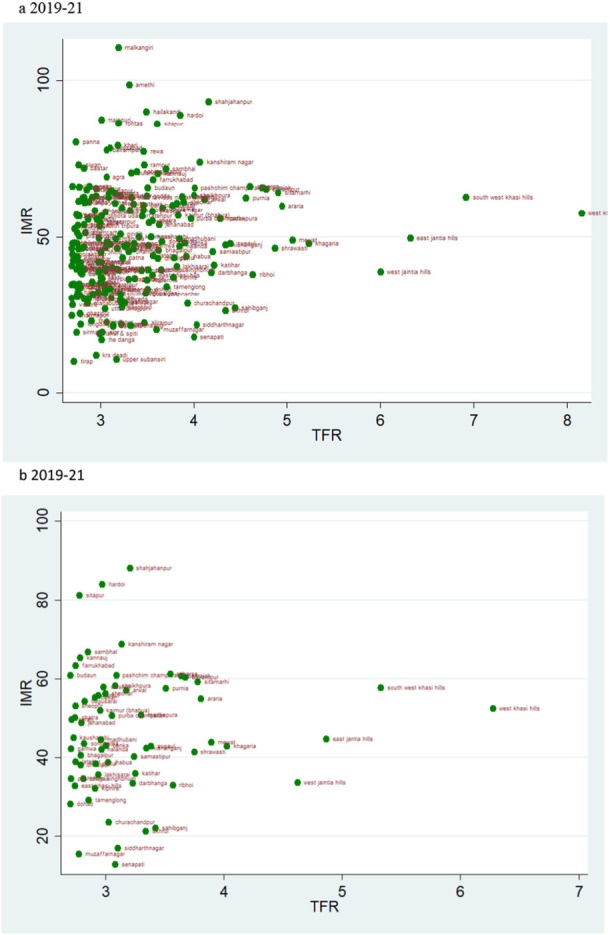
a, b: Scatter Plot of District‐Level Infant Mortality Rates (IMR) and Total Fertility Rates (TFR) for Districts with TFR above 2.7, 2015–16 to 2019–21.

The scatter plot suggests a trend of positive change in the rate of IMR over two time periods. If we evaluate the magnitude of infant mortality in the group with TFR less than 1·6, we see a favourable transitional trend, with more districts displaying a lower magnitude of IMR in 2019–21 (Figure 8a,b). Most districts had TFRs between 1.6 and 2.1, with mortality rates under 25 over the two time periods (Figure 9a,b). For TFR between 2.1 and 2.7, the number of districts with IMR over 80 has increased over the two time periods (Figure 10a,b). Figure 11a,b depict IMR in districts with more than 2.7 TFR. Most of the districts with TFR above 2.7 had IMR below 50 for both survey periods (Figures 11a,b). We observed a random trend in the infant mortality rate in the district groups, with TFRs ranging from 1.6 to 2.1 and 2.1 to 2.7. We can see the difference by comparing the scatter plot of 1.6–2.1 in 2019–21 to 2.1–2.7 in 2015–2016 (Figures 9a and 10b). Since, these two groups have about similar numbers of districts, we may expect a shift between them over time. Furthermore, when we look at the magnitude of child mortality rates, we observe that rates primarily fall between 20 and 40.

**Figure 12:**
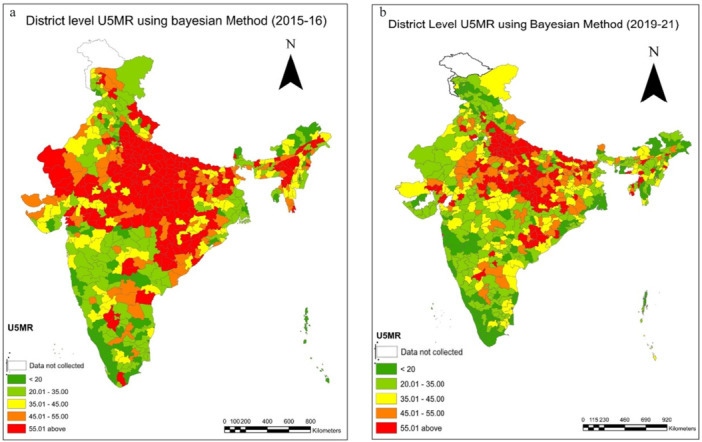
a, b: District level estimates of Under 5 Mortality Rates(U5MR) using Bayesian Method during 2015–16 to 2019–21.

### 4.5 TFR Vs U5MR

**Figure 13:**
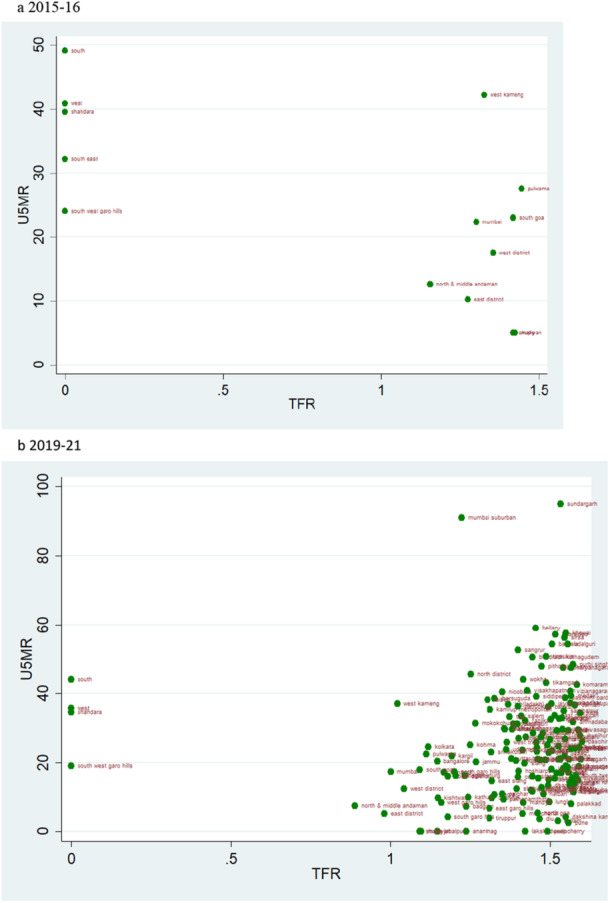
a, b: Scatter Plot of District‐Level Under‐Five Mortality Rates (U5MR) and Total Fertility Rates (TFR) for Districts with TFR Below 1.6, 2015–16 to 2019–21.

**Figure 14:**
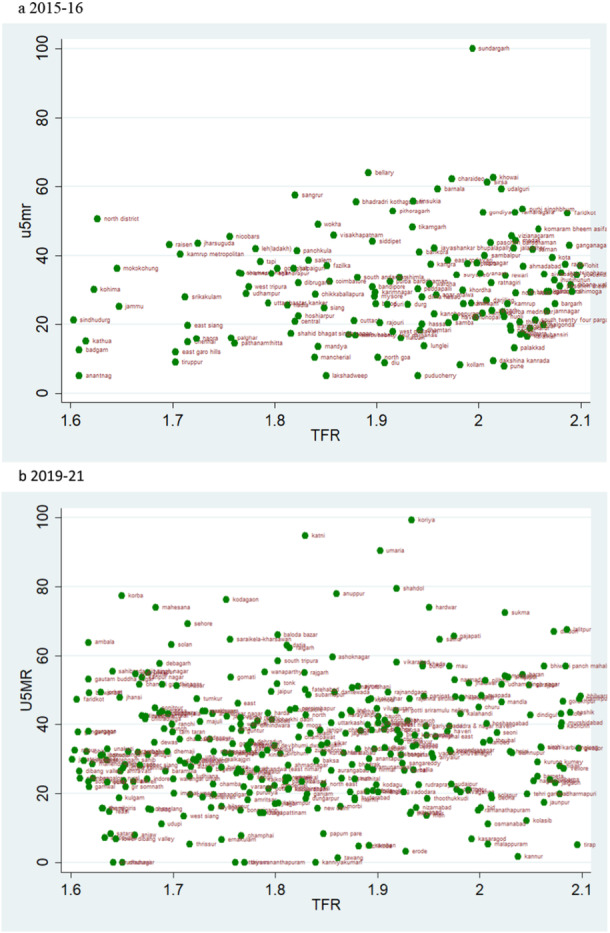
a, b: Scatter Plot of District‐Level Under‐Five Mortality Rates (U5MR) and Total Fertility Rates (TFR) for TFR Between 1.6 and 2.1, 2015–16 to 2019–21.

**Figure 15:**
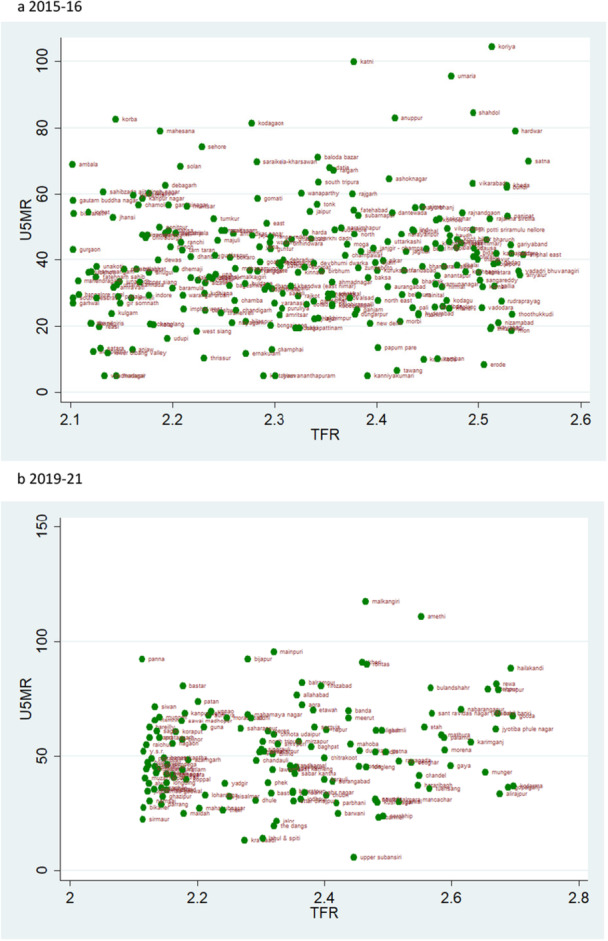
a, b: Scatter Plot of District‐Level Under‐Five Mortality Rates (U5MR) and Total Fertility Rates (TFR) for TFR Between 2.1 and 2.7, 2015–16 to 2019–21.

**Figure 16:**
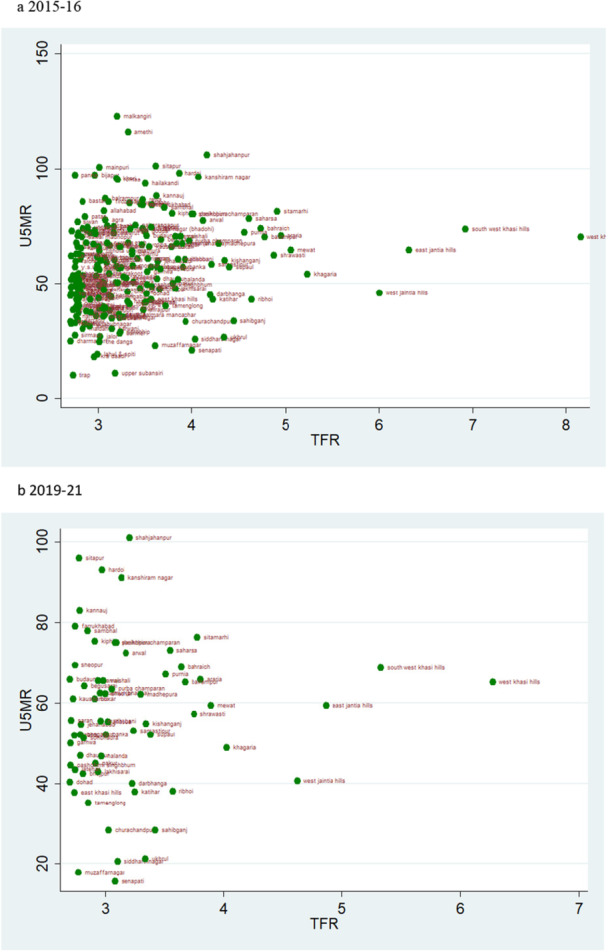
a, b: Scatter Plot of District‐Level Under‐Five Mortality Rates (U5MR) and Total Fertility Rates (TFR) for Districts with TFR Above 2.7, 2015–16 to 2019–21.

A pattern of change in the rate of U5MR across two time periods for various categories of TFR is also depicted by the scatter plot. A similar pattern as of NMR and IMR can be observed when TFR is lower than 1.6 i.e., there are more number of districts in 2019–21 than in 2015–16, but the magnitude of U5MR is lower (Figure 13a,b). The U5MR in the groups with TFRs ranging from 1.6 to 2.1 and 2.1 to 2.7 showed a random pattern (Figures 14a,b and 15a,b). The change may be seen by comparing the scatter plot of 1.6–2.1 in 2019–21 to 2.1–2.7 in 2015–2016 regarding reducing magnitude of mortality rate (Figures 14a and 15b). The scatter plot of TFR > 2.7 with U5MR for the year 2019–21 reveals comparatively few number of districts as compared to 2015‐16 (Figure 16a,b). We can readily determine that there is a positive correlation between TFR and child mortality rates. Moreover, we observed a change in all groups over two periods, with lower TFR and better mortality rates across districts.

## 5 Discussion

The research in this study is based on fertility and child mortality estimations from NFHS surveys conducted between 2015‐16 and 2019‐21. The fertility transition across Indian states should exhibit a simultaneity effect on child mortality. This research clearly illustrates, using maps, that the general trend in TFR, NMR, IMR and U5MR is decreasing and India is heading in the right direction. TFR and IMR is already significantly lower in less densely inhabited districts. Nonetheless, significant work remains to be done in densely populated districts, where mortality drop has been slower than predicted, despite several policy interventions.

Between 2015‐16 and 2019‐21, India recorded substantial reductions in fertility and child mortality rates, accompanied by a gradual narrowing of the performance gap between the best‐ and worst‐performing districts. Despite notable improvements in child mortality and fertility rates in recent years, substantial gaps persist, particularly in underperforming districts that continue to stand out as outliers. The findings carry important policy implications. Targeted and adaptive strategies are needed to address persistently high fertility and child mortality in these districts, with a particular emphasis on reaching lower socioeconomic groups. Irrespective of socioeconomic differentials, the evidence indicates that states such as Madhya Pradesh, Uttar Pradesh, Chhattisgarh, and Bihar benefitted considerably from an accelerated scale‐up of interventions aimed at reducing child mortality at the population level.

Child mortality estimates can effectively segregate the particular districts of the states and address them through targeted programmes and policies. Considering the spatial location of the better‐performing states in southern India, a lower value of child mortality outcomes is observed. However, the presence of outliers in the districts distorts the approach towards development at the community level. Kerala, despite achieving almost negligible mortality rate among children, few districts have been seen to be relatively higher than others. The primary reason for higher child mortality, especially neonatal mortality, is genetic disorders, higher maternal mortality rate, poor behavioural outcomes, etc. The consanguineous marriage followed in particular communities can result in congenital disabilities and poor survival in the early life years.

However, the programmatic approach of providing care through crèche, nutritional support at the community, and other developmental initiatives at the community level could assist in improving child malnourishment and motivates work participation. Grassroots institutions like Village Education Committees and Mothers’ Committees in Andhra Pradesh have promoted parental participation in maternal and child health services and have induced better community‐level participation in accessing health services [21]. Improved performance monitoring, bestowing financial autonomy to hospitals, centralising and awarding states, and empowering the authority to procure drugs are some of the effective measures taken in Rajasthan and Andhra Pradesh that have led to their speedy development. A growing body of literature holds evidence that the decentralisation of management boards at health facilities and fiscal decentralisation has helped in reducing infant mortality and fertility levels in many rural districts of India [22, 23].

The study also highlights the community's involvement in improving child survival in India. However, these practices remained classified by region, religion, and other socio‐economic groups. In the northern states, clusters of child malnourishment and mortality are concentrated in several districts; effective involvement of local governments could lead to greater development in these localities. Due to rugged terrain, the north‐eastern states need more infrastructure at primary healthcare services. However, the scenario has improved between 2015‐16 and 2019–21. Only one‐fifth of the rural women access three or more Antenatal Care (ANC) in Bihar and Nagaland, which explains the structural failure of the healthcare system [24].

In Kerala, districts like Alappuzha, Wayanad, and Kollam are showing relatively higher level of child mortality rates, despite overall strong health indicators, factors like congenital anomalies, consanguineous marriages in certain communities, and higher maternal age at childbirth may contribute to localized higher child mortality rates. The possibility that stillbirths are reported as neonatal deaths cannot be ruled out in large scale surveys. Further in‐depth research on these issues is required. In Uttar Pradesh, persistent issues such as poor maternal healthcare access, lower health infrastructure coverage, socio‐cultural practices surrounding early marriage and childbirth, and nutritional deprivation remain key contributors to higher child mortality, even in districts where fertility has declined.

Being a country that started the policies for family planning well ahead of other nations with similar demographic characteristics, India needs to catch up on several demographic and socio‐economic aspects. Recently, reshaping the health and population policies has taken up approaches that prioritize neonatal care to dampen the burden of child mortality. Policies such as the National Health Mission (NHM) and the Pradhan Mantri's Overarching Scheme for Holistic Nourishment (POSHAN Abhiyaan) have emphasised facility‐based newborn care, strengthening the capacity of first referral units, and training Accredited Social Health Activists (ASHAs) for home‐based newborn care, with a primary focus on rural areas. The northern states like Bihar, Uttar Pradesh, Madhya Pradesh, etc., require district‐specific plans for aversion of child mortality so that every facet of the primary healthcare norms is achieved. The involvement of grassroots‐level workers, when provided with proper training, has been recognised as an effective way to bridge the gap left by shortages of trained doctors and paramedics [25]. Therefore, current policies must adopt multidimensional developmental approach aimed at improving the two most crucial demographic parameters: fertility and mortality. It is also essential to enhance public financing in the healthcare sector. Lack of accessibility in the government health sectors increases out‐of‐pocket expenditure and the likelihood of poor child survival. The lower socio‐economic groups benefit from public transfers more than private transfers [26]. Thus, resource allocation and effective utilisation must be evoked to uphold development in the country's most deprived regions. To comprehend the fertility outcome of the population in the backward states of India, it is of utmost importance to strengthen the programmes and policies that are acceptable to and supported by the community.

Enhanced maternal care such as increased antenatal visits, skilled birth attendance, and institutional deliveries reduces maternal and neonatal complications, lowering child mortality rates. Simultaneously, improved child health services, immunization coverage, and postnatal care increase child survival, which, in turn, reduces the demand for additional births as a replacement strategy. Access to family planning counselling integrated within maternal health services further enables women to space or limit births, contributing to fertility decline.

In the wake of the 2013 Reproductive, Maternal, New‐born, Child and Adolescent Health (RMNCH + A) strategic framework and the Call to Action for Child Survival and Development, several policy initiatives were introduced in India. The RMNCH+ plan is a continuum‐of‐care‐based framework that defines integrated service packages for distinct life stages. The 4Ds ‐birth defects, deficiencies, diseases, and developmental delays, have been introduced more recently, along with web‐enabled tracking of expectant mothers to ensure antenatal, intrapartum, and postpartum care.The Janani Shishu Suraksha Karyakram guarantees all expectant mothers giving birth in public health facilities the right to free delivery, including a caesarean section and accessible transportation. Throughout the years, India has made considerable progress in enhancing child health and lowering fertility rates, but more work remains to be carried out. Several social, economic, and environmental elements that impact children's health outcomes provide one of India's most significant obstacles to improving child health. However, the pattern of child health in India may be improved with targeted interventions and policies. In India, key measures to improve child health outcomes include increasing the number of healthcare facilities in rural and remote areas, enhancing the standard of healthcare services, and ensuring that all children have access to necessary medicines and vaccinations.

## 6 Conclusions

This study gives an overview of India's child mortality outcome in coherence with fertility outcomes. It can be concluded from the study that the fertility and child mortality changes are coherence to each other for most of the districts over two survey periods, and any deviance could be attributed to a failure in the programmatic approaches and insufficient attention to the complex socio‐cultural differences across regions. Nevertheless, results have noted a mixed finding while assessing the relationship between fertility and mortality rates. In most districts, mortality rates declined with a decline in fertility rates, whereas, in a few districts, lower fertility rates were found with higher mortality rates. This paradox could be due to a change in the study setting as different districts are at differing level of socio‐economic status. Furthermore, it might be possible that in certain districts, the fall in fertility rates might have been faster than the fall in mortality rates. Specific studies will be carried out in that area to understand this paradox. States whose fertility is converging need to follow sufficiently similar pathway for development due to the individual and environment‐specific components. Policies should bolster institutional delivery, prevent child marriage and early pregnancy, and provide proper nutritional programmes. Primary investigations at the village level should be carried out to understand the problems in the effective implementation of the already functioning policies. Policies should be empathetic and encourage women to exercise their reproductive rights rather than force them to adopt any practices. The fertility transition across Indian states should exhibit a simultaneity effect on child mortality. which are not suggested. Active community participation should be promoted and expanded to change the attitude and behaviour of the population.

## Author Contributions

L.K.D. and M.B. conceptualized the study. L.K.D., S.C. S.J. and P.D.were responsible for writing the original draft. L.K.D., S.J., S.C., P.A. and P.D. were involved with formal analysis of the data. All the authors have read and approved the manuscript.

## Funding

The authors received no specific funding for this work.

## Ethics Statement

This analysis is based on a secondary dataset with no identifiable information on the survey participants. This dataset is available in the public domain for research use hence no approval was required from any institutional review board as there is no question of human subject protection arising in this case.

## Conflicts of Interest

The authors declare no conflicts of interest.

## Permission to Reproduce Material From Other Sources

Data used in this study is publicly available and can be accessed from the DHS programme website (https://dhsprogram.com/methodology/survey/survey-display-355.cfm).

## Transparency Statement

The lead author Laxmi Kant Dwivedi affirms that this manuscript is an honest, accurate, and transparent account of the study being reported; that no important aspects of the study have been omitted; and that any discrepancies from the study as planned (and, if relevant, registered) have been explained.

## Supporting information


**Table 5:** Bayesian estimates of district‐level neonatal mortality rate (NMR), infant mortality rate (IMR), under‐five mortality rate (U5MR), and total fertility rate (TFR) for India, 2015–16. **Table 6:** Bayesian estimates of district‐level neonatal mortality rate (NMR), infant mortality rate (IMR), under‐five mortality rate (U5MR), and total fertility rate (TFR) for India, 2019‐21.

## Data Availability

The data is available online on the website and can be downloaded. International Institute for Population Sciences was the nodal agency for NFHS‐5; therefore, being the faculty and students of this institute, we have accessed the data from the institute's datacentre. Data available in DHS site https://dhsprogram.com/data/dataset_admin/login_main.cfm.
